# Oral Health Interventions in Children With Autism Spectrum Disorder: A Systematic Review and Meta‐Analysis of Interventional Studies

**DOI:** 10.1002/cre2.70258

**Published:** 2025-12-29

**Authors:** Iman Rajaei, Azadeh Babaei, Shirin Djalalinia, Mostafa Qorbani

**Affiliations:** ^1^ Student Research Committee Alborz University of Medical Sciences Karaj Iran; ^2^ Community Oral Health Department, School of Dentistry Alborz University of Medical Sciences Karaj Iran; ^3^ Development of Research & Technology Center, Deputy of Research and Technology Ministry of Health and Medical Education Tehran Iran; ^4^ Non‐communicable Diseases Research Center Alborz University of Medical Sciences Karaj Iran; ^5^ Chronic Diseases Research Center, Endocrinology and Metabolism Population Sciences Institute Tehran University of Medical Sciences Tehran Iran

**Keywords:** autism spectrum disorder, interventions, oral health

## Abstract

**Objectives:**

Children with autism spectrum disorder (ASD) exhibit a higher prevalence of oral health diseases. These oral health issues significantly impair quality of life and increase caregiver burden. Despite emerging interventions like specialized behavioral guidance and caregiver training, a comprehensive evaluation of their effectiveness remains absent. This systematic review and meta‐analysis was designed to pool the effectiveness of Oral health interventions in children with ASD.

**Materials and Methods:**

In this systematic review, some international databases, including PubMed, ISI/WOS, CENTRAL, and Scopus, were searched via appropriate keywords until January 1, 2025. All clinical trials that assessed the effect of interventions on oral health indices including Simplified Oral Hygiene Index (OHI‐S), plaque index (PI), and gingival index (GI) without any language or time restrictions were included in the study. Search strategy process, screening, data extraction, and quality assessment were performed by two experts independently. Heterogeneity between studies was assessed using I^2^ and Q‐Cochrane test. Random effect meta‐analysis was performed to pool the standardized mean difference (SMD) with 95% confidence intervals (CIs).

**Results:**

Overall, 27 studies with 1918 participants were included in this study. Interventions are categorized into the educational (visual, verbal, booklet/leaflet) and procedural approaches. Random effect meta‐analysis showed significant improvements for PI (SMD = −0.73, 95% CI: [−1.02 to −0.44]) and OHI‐S (SMD = −1.44, 95% CI: [−2.79 to −0.08]), with video interventions notably effective for PI (SMD = −0.69, 95% CI: [−1.2 to −0.14]). The GI index also improved (SMD = −0.74, 95% CI: [−1.34 to −0.14]).

**Conclusions:**

Visual pedagogy, particularly videos, and parental involvement significantly improved oral health in children with ASD. Video‐based interventions should be added to traditional methods such as verbal or picture‐based approaches to improve oral health interventions in ASD.

**Abbreviations**
CBCL: child behavior checklist
CI: confidence interval
CI: confidence interval
DI: Debris index
DMFT/dmft: decayed, missing due to caries, and filled permanent/deciduous teeth
GI: gingival index
NRSI: nonrandomized studies of interventions
N/A: not applicable
OHI‐S: Simplified Oral Hygiene Index Score
PAIR: Picture Assisted Illustration Reinforcement
PCR: polymerase chain reaction
PECS: Picture Exchange Communication System
PI: Plaque index
RCT: randomized clinical trial
SMD: standardized mean difference
TBVP: Tooth Brushing Visual Pedagogy
VM: video modeling
VP: visual pedagogy

## 1 Introduction

Autism spectrum disorder (ASD) is a neurodevelopmental condition characterized by challenges in social interaction, communication, and restricted or repetitive behaviors (American Psychiatric Association D, Association AP 2013). The prevalence of ASD has risen significantly in recent decades, with current estimates showing that 1 in 100 children around the world (Zeidan et al. 2022). Along with the main symptoms of ASD, individuals frequently face co‐occurring health issues that affect their overall well‐being. Poor oral health is an essential yet underexplored concern among these. Research consistently demonstrates that children and adults with ASD present higher rates of dental diseases, including caries, gingivitis, and periodontitis, compared to their neurotypical peers (Du et al. 2022a; Loo et al. 2009). These differences are attributed to a complex interaction of factors, including heightened sensory sensitivities, behavioral challenges, communication difficulties, medication side effects, and motor skill impairments (Stein et al. 2011; Friedlander et al. 2006; Marshall et al. 2007).

Poor oral hygiene in individuals with ASD significantly reduces their quality of life, contributing to pain, nutritional deficits, school absenteeism, and social difficulties (Jaber 2011). This population's access to personalized dental care remains limited, worsening health inequities (Weil and Inglehart 2010), which refer to disparities in health outcomes and access to healthcare services between different population groups (Filipe et al. 2024). Caregivers, primarily responsible for managing oral health routines and securing dental treatment, report substantial stress and logistical barriers, including a lack of dental professionals trained in ASD‐specific care (Lai et al. 2012; Casamassimo et al. 2004). Existing studies have documented the prevalence of oral health issues in this population (Da Silva et al. 2017). However, there is a paucity of evidence‐based interventions designed to address these challenges effectively.

Emerging research highlights promising approaches, such as specialized behavioral guidance, visual supports, and caregiver training, to improve oral health outcomes in children with ASD (Pai Khot et al. 2023; Fenning et al. 2022; Lu and Liu 2022). Given the complex needs of this group, a systematic review is urgently needed to compile all available data and assess the quality and outcomes of personalized oral health interventions. Such an analysis could inform evidence‐based guidelines for dental practitioners, reduce caregiver burden, and bridge the gap in oral health equity for policymakers. This study addresses this critical research priority by presenting a systematic review of specialized interventions to enhance oral health care delivery and outcomes for children with ASD.

## 2 Materials and Methods

The present study is a comprehensive review of oral health interventions in children with ASD. We systematically searched and analyzed all available related data without any limitation of publication language or time of publication. We aim to follow the latest guidelines of Preferred Reporting Items for Systematic Reviews and Meta‐Analyses (PRISMA 2020) (Moher et al. 2015; Preferred reporting items for systematic review and meta‐analysis protocols (PRISMA‐P) 2015: elaboration and explanation 2016; Shamseer et al. 2015; Page et al. 2021).

### 2.1 Inclusion and Exclusion Criteria

#### 2.1.1 Types of Studies

All interventional studies, including randomized/nonrandomized clinical trials and quasi‐experimental studies with or without comparator arms, were included. There was no restriction on the time and language of publications.

#### 2.1.2 Types of Participants

Children and adolescents under 20 years old who had a definitive diagnosis of ASD, based on published reports, were considered for inclusion.

#### 2.1.3 Types of Interventions

Our study, regardless of the target group (patient‐based interventions or dentists/teachers/parents/caregivers‐based interventions), contents and channels (training, preventive techniques, procedures, behavior changing modules, etc.), methods (mass media programs, video modeling, picture exchange communication system, verbal, book/booklet/pamphlet/leaflet, etc.) and scope of implementation (individual based or school/community based), is solely focused on interventions aimed at improving oral health or behaviors or procedures which cause this result, in children with ASD. We assessed outputs, outcomes, and impacts to ensure a comprehensive understanding of the interventions' effectiveness.

#### 2.1.4 Types of Outcome Measures

To assess the outputs, outcomes, and impacts of oral health interventions, all quantitative indicators (DMFT/dmft, OHI‐S, PI, GI, and etc.), as well as mixed‐method indicators (feasibility, acceptability, quality adjusted life years (QALY), autonomy, and etc.), were extracted and analyzed.

### 2.2 Data Sources and Search Strategies

The main root of the systematic search strategy, developed based on two main components: “oral health interventions” and “autism spectrum disorder,” was completed through the process of implementation in each of the relevant databases based on their specific tools and techniques (MeSH term, Emtree, and complementary keywords).

On January 1, 2025, searches ran through the main relevant international databases of PubMed/Medline, ISI/WOS, CENTRAL, and Scopus (Table 1). Moreover, the Google Scholar search engine was checked to ensure better access to the full text of the found papers.

**Table 1:** Search strategy.

PubMed
(((“Autism Spectrum Disorder”[Mesh]) OR (“Autistic Disorder”[Mesh])) OR (Autism[Title/Abstract])) AND ((((“Oral Health”[Mesh]) OR (“Oral Hygiene”[Mesh])) OR (dental[Title/Abstract])) OR ((“oral health”[Title/Abstract]) OR (“oral hygiene”[Title/Abstract])))
Scopus
((TITLE‐ABS‐KEY (autism) OR TITLE‐ABS‐KEY (“Autistic Disorder”))) AND ((TITLE‐ABS‐KEY (“Oral Health”) OR TITLE‐ABS‐KEY (“Oral Hygiene”) OR TITLE‐ABS‐KEY (dental))) AND (LIMIT‐TO (SUBJAREA, “MEDI”) OR LIMIT‐TO (SUBJAREA, “DENT”) OR LIMIT‐TO (SUBJAREA, “HEAL”) OR LIMIT‐TO (SUBJAREA, “NURS”)) AND (LIMIT‐TO (DOCTYPE, “ar”) OR LIMIT‐TO (DOCTYPE, “re”)) AND (LIMIT‐TO (SRCTYPE, “j”))
ISI/WoS
**“Autism Spectrum Disorder”** (Topic) or **“Autistic Disorder”** (Topic) or **Autism** (Topic)
**“Oral Health”** (Topic) or **“Oral Hygiene”** (Topic) or **dental** (Topic)
#1 AND #2

### 2.3 Study Selection

The search results were imported into EndNote 21 software for better data management. Articles were retrieved for quality appraisal after the three steps of refinement for titles, abstracts, and full‐text relevancy.

### 2.4 Risk of Bias Assessment

The quality of the articles was assessed based on the National Institute of Health (NIH) Critical Appraisal Checklist for quasi‐experimental studies (National Heart, Lung, and Blood Institute 2014), the Risk of Bias In Non‐randomized Studies of Interventions (ROBINS‐I) assessment tool for non‐randomized clinical trial studies (Sterne et al. 2016), and the Revised Cochrane risk‐of‐bias tool for randomized trials (RoB 2) for randomized clinical trial studies (Sterne et al. 2019). The GRADE framework (Schünemann et al. 2013; Guyatt 2009) was used to assess the strength of the evidence.

### 2.5 Data Extraction

Data were extracted using a predefined check list recording citation, including (1) bibliographic specification (first author and publication year); (2) methodological criteria (place of study, study design, sample size, type and description of interventions and control groups (if available) and indices), and (3) outcome measures (quantitative and mixed‐method results before and after interventions). The first and the last measurements were reported.

The entire process, from systematic search to final data extraction and analysis, was followed and completed independently by two researchers (Kappa statistic for agreement for quality assessment: 0.92). The main investigator resolved any discrepancies.

### 2.6 Data Synthesis and Meta‐Analysis

Statistical analyses were performed using STATA 17 software. Standard mean difference (SMD) with confidence intervals (CIs) was used to assess the pool effect of the intervention on outcomes. The heterogeneity between studies was evaluated by I^2^ statistic and Q Cochrane chi‐square‐based test (Sterne et al. 2019). *p* Values of the chi‐square test of heterogeneity were considered statistically significant at 0.1. A stratified meta‐analysis was performed according to the type of outcomes, study design, and intervention. Sensitivity analysis was used to estimate effect of exclusion of some studies from meta‐analysis on pooled estimates of SMD with 95% CIs.

Potential publication bias was assessed using Egger's tests, and the results of Egger's tests were statistically significant at *p* < 0.1 (Egger et al. 1997). If there was publication bias, the “trim‐and‐fill” method was used to adjust and correct the publication bias (Shi and Lin 2019).

### 2.7 Ethical Considerations

The present paper was extracted from the DDS student thesis, which has been approved by ethics committee of Alborz University of Medical Sciences (ethics code: IR. ABZUMS. REC.1403.159).

We also registered and received approval from Prospero; PROSPERO Registration number: CRD42024514467.

All included studies that have influenced the reported outcomes have been cited within this article and will be cited in all of the publications extracted from this study.

For further information required, the corresponding authors were contacted.

## 3 Results

### 3.1 Search Results

**Figure 1:**
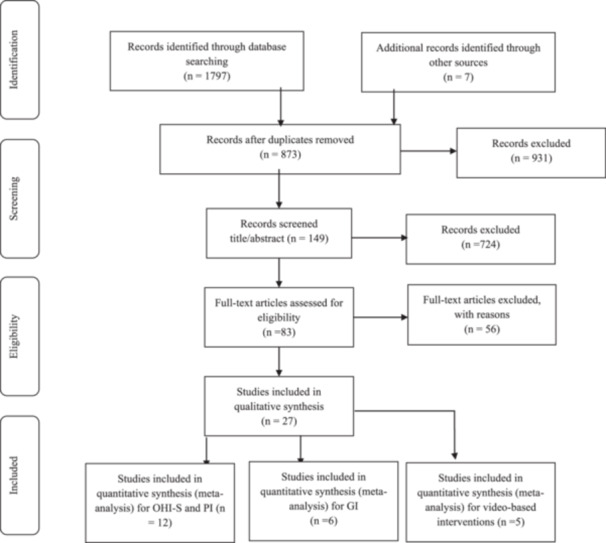
Flowchart of the number of studies selected for the meta‐analysis.

From the 1797 studies of the initial search, 931 were duplicates. After addition 7 records identified through other sources, 873 articles were evaluated, and 149 were considered relevant based on the title screening, and 83 were considered relevant based on abstract screening. The remaining articles' full text then, were assessed and evaluated for eligibility the inclusion criteria. Finally, 27 articles met the inclusion criteria. All included studies met quality assessment score. This process is illustrated in Figure 1.

### 3.2 General Characteristics

These studies were conducted worldwide (United States of America, Sweden, Hong Kong, Saudi Arabia, Egypt, China, India, Iran, Jordan, Malaysia, Italy, Thailand, Bulgaria, and France).

Regarding the study design, 12 were RCTs, 2 were non‐randomized studies, and 13 were quasi‐experimental studies. The total number of participants was 1918 with an age range of 2 to 19 years. These data, alongside other study characteristics, are presented in Table 2.

**Table 2:** General characteristics of the studies.

First author, year	Country	Study design	Sample size	Type of intervention		Type of control	
				Name	Description	Name	Description
(Vajawat et al. 2015)	India	RCT	I: 20 C: 20	Powered toothbrush	Colgate 360° sonic power toothbrush generating 20,000 oscillations per minute	Manual toothbrush	Colgate 360° toothbrush
(Gandhi et al.^2024^)	USA	RCT	I:14 C: 11	VM	Daily email links to a YouTube video showing a young girl demonstrating and describing toothbrushing steps	Social story	Daily email links to a pictorial guide describing toothbrushing steps
(Popple et al. 2016)	USA	RCT	I: 9 C: 9	VM	Narrative video and closed captions; After 3 weeks, participants received a YouTube link for continued access	Passive video	Included abstract visual content (colorful fractal images with music) still received email reminders but no brushing instruction.
(Du et al. 2022b)	Hong Kong	RCT	I: 119 C: 103	TBVP	A flip‐chart storybook and an associated DVD contained real‐life photos of a boy demonstrating toothbrushing, with subtitles and background music	Conventional oral health instruction	Standard oral health education using tooth models
(Aljubour et al. 2022)	Saudi Arabia	RCT	I: 32 C: 32	Culturally adapted dental visual aids	Culturally adapted dental visual aids that were specially designed and developed for this study by an artist who used images based on photographs of the actual pediatric dentistry clinic setting	Regular dental visual aids	Regular dental visual aids extracted from the website www.ageofautism.com
(Ibrahim et al. 2020)	Egypt	Quasi‐experimental	I: 60	Health education for mothers	Sessions focusing on the importance and impact of oral health on the quality of life, included interactive lectures, video presentations, and booklets	N/A	N/A
(Mah et al. 2023)	Malaysia	Quasi‐experimental	I: 32	Oral health care module	A booklet with oral health care guidelines, a modified tooth‐brushing technique and a poster	N/A	N/A
(Al‐Batayneh et al. 2020)	Jordan	Quasi‐experimental	I: 37	PECS	Two sets of PECS cards for visual guidance on tooth brushing, one set with smaller pictures and the other with larger pictures and written instructions.	N/A	N/A
(Sallam et al. 2013)	Egypt	RCT	I1: 12 I2: 12 C: 12	Group B: pictorial activity schedule group C: VM	Group B: A series of colored pictures showing the tooth brushing steps in a sequence Group C: A demonstrated tooth brushing steps in a real setting with verbal instructions.	Group A: Jaw Demonstration Modeling	A model representing the Fones brushing technique on the upper and lower jaw with visual instructions
(Krishnan et al. 2021)	India	NRSI	I: 30 C: 30	VP	Visual cards depicting the Modified Bass tooth brushing technique	Mobile application brush up	Featuring a 3D animation video for tooth brushing with interactive elements, including a character that teaches tooth brushing techniques.
(Pai Khot et al. 2023)	India	RCT	I: 30 C: 30	PAIR system	sequential pictures with instructions to demonstrate correct oral hygiene tasks	Conventional verbal oral health education	verbal oral health education including demonstrations of the Fones method but without visual aids
(Du et al. 2021)	Hong Kong	Quasi‐experimental	I: 122	TBVP	A flip‐chart containing 13 photographs and a training DVD	N/A	N/A
(Mahajan et al. 2023)	India	Quasi‐experimental	I: 100	Smartphone VP	A compatible Android and Apple smartphone's video demonstrating the Fones tooth brushing technique	N/A	N/A
(Pawar et al. 2022)	India	RCT	I: 20 C: 20	Powered toothbrush	Colgate 360° sonic control toothbrush, generating 20,000 high‐energy sonic oscillations per minute	Manual toothbrush	Manual toothbrushes
(Carli et al. 2022)	Italy	Quasi‐experimental	I: 100	Preventive Oral Health Program	A prevention program conducted through five structured visits, incorporating behavioral strategies and interactive digital supports. Parents were involved in home training using digital videos and interactive PDFs	N/A	N/A
(Doichinova et al. 2019)	Bulgaria	Quasi‐experimental	I: 30	PECS	Sequence of actions involved in brushing, which were presented in a strict order to represent the steps in maintaining oral hygiene	N/A	N/A
(Fenning et al. 2022)	USA	RCT	I: 60 C: 59	Parent training	Home visit, dental office coach, phone booster sessions, social stories, video modeling, and visual schedules	Psychoeducational dental toolkit	Physical Health Dental Toolkit No behavior training or in‐person coaching was provided
(Shalabi et al. 2022)	Egypt	RCT	I: 25 C: 25	VM	Animated characters demonstrating various toothbrushing techniques	PECS	A series of 12 pictures illustrating the steps of toothbrushing
(Renuka et al. 2022)	India	Quasi‐experimental	I: 30	PECS	Pictures of dental operatory	N/A	N/A
(Piraneh et al. 2023)	Iran	NRSI	I: 79 C: 58	VM	A video, containing 23 small stages of the Fones technique	Social story	A social story with images and sentences explaining the 23 stages of the Fones technique
(Zhou et al. 2020)	China	RCT	I: 156 C: 150	Social story	Social stories through visual and verbal integration	Standard leaflet	Standard leaflets without the structured social story approach
(Pilebro and Bäckman 2005)	Sweden	Quasi‐experimental	I: 14	VP	A series of pictures depicting structured steps of tooth brushing	N/A	N/A
(Lefer et al. 2018)	France	Quasi‐experimental	I: 52	Tablet‐çATED App	The çATED application includes visual guidance, verbal prompts, and behavioral reinforcement	N/A	N/A
(Smutkeeree et al. 2020)	Thailand	Quasi‐experimental	I: 30	VP	A set of drawing‐based visual pedagogy materials include picture sequence illustrating the steps of toothbrushing	N/A	N/A
(Lopez Cazaux et al. 2019)	France	Quasi‐experimental	I: 52	iPad‐ çATED App	The çATED application includes visual guidance, verbal prompts, and behavioral reinforcement	N/A	N/A
(Chawla and Goswami 2021)	India	Quasi‐experimental	I: 10	VM	A customized instructional video for the Fones technique	N/A	N/A
(Ramassamy et al. 2019)	India	RCT	I: 36 C: 36	Yoga therapy as an adjunct to VP and VM	Visual pedagogy and video modeling, but also participated in daily 1‐h yoga	VP and VM	Pictures illustrating toothbrushing steps and video modeling

Abbreviations: N/A, not applicable; NRSI, nonrandomized studies of interventions; PAIR, Picture Assisted Illustration Reinforcement; PECS, Picture Exchange Communication System; RCT, randomized clinical trial; TBVP, Tooth Brushing Visual Pedagogy; VM, video modeling; VP, visual pedagogy.

First study was done in 2005 and the last one was done in 2024.

Most of these studies were from India (eight studies). The largest sample size was from Hong Kong with 306 participants (Du et al. 2022b), and the smallest was from India with 10 participants (Chawla and Goswami 2021).

There were 16 studies for interventions in children with ASD and 5 studies for interventions in their caregivers/parents/teachers and 6 studies for both of them.

These general characteristics of included studies for oral health interventions for children with ASD are shown in Table 2.

### 3.3 Mixed‐Method Synthesis

In the comprehensive literature review, a wide range of interventions was identified for children with ASD, their parents/caregivers/teachers, or both of these groups (Table 3). These interventions were categorized into two content areas, education and procedure. The oral health educational content was thorough, covering three channels (Visual, Verbal, and booklet/Leaflet). The Visual channel was particularly comprehensive, offering a range of options, including Images/Pictures, Videos, and Email/Application/mobile program.

**Table 3:** Main characteristics of the included studies.

First author, year	Outcome	Index	Result				Summary of findings
			Intervention group		Control group		
			Before	After	Before	After	
(Vajawat et al. 2015)	Oral hygiene	PI	1.03 ± 0.37	0.71 ± 0.30	1.03 ± 0.26	0.93 ± 0.29	A statistically significant improvement
		GI	0.75 ± 0.29	0.49 ± 0.25	0.81 ± 0.23	0.65 ± 0.20	
		Detection of red complex organisms using PCR					No statistically significant reduction
(Gandhi et al. 2024),	Oral hygiene	Modified PI	1.30 ± 0.19	0.62 ± 0.13	1.20 ± 0.13	0.70 ± 0.12	A statistically significant improvement
		Modified GI	1.15 ± 0.19	0.56 ± 0.11	1.18 ± 0.13	0.78 ± 0.13	
	Caregiver survey responses	Acceptance of toothbrushing	57.1%	50%	54.5%	36.4%	VM group showed greater acceptance of daily oral hygiene compared to control group. Improved child behaviors towards daily oral hygiene (acceptance and ease of toothbrushing) in the VM compared to the TSS group
		Ease of toothbrushing	28.6%	50%	72.7%	63.6%	
(Popple et al. 2016)	Oral hygiene	PI	1.78 ± 0.62	0.38 ± 0.43	1.75 ± 0.83	1.20 ± 1.05	A statistically significant improvement in both groups
	Parental & children engagement and motivation						Some parents reported children reciting the video prompts while brushing, or the video reinforced brushing routines at home or improvements, possibly due to the structured reminders to brush.
(Du et al. 2022b)	Toothbru‐shing skill	Performance in the toothbrushing	1.88 ± 1.40	2.87 ± 1.57	1.59 ± 1.49	2.22 ± 1.49	TBVP significantly improved preschoolers' toothbrushing skills compared to conventional oral health instruction.
		Rating of Good toothbrushing task	32.8%	58.8%	17.5%	42.7%	
(Aljubour et al. 2022)	Oral hygiene	PI	1.445 ± 0.56	0.45 ± 0.50	1.724 ± 0.69	1.10 ± 0.68	Both groups showed significant improvement
(Ibrahim et al. 2020)	Knowledge	Total knowledge score	10.716 ± 2.64	20.450 ± 2.07	N/A	N/A	Significant improvements in mothers' knowledge and practices regarding oral health care for their children
	Practice	Total Practice Score	3.62 ± 4.09	14.97 ± 5.78			
	Quality of life	Total Quality of Life Scores	41.50 ± 3.74	37.40 ± 3.92	N/A	N/A	The quality of life for autistic children improved significantly.
(Mah et al. 2023)	Ability	Modified Checklist for Autism in Toddlers (M‐CHAT)					Improvements in children's tooth‐brushing cooperation and a reduction in behavior problems related to brushing
(Al‐Batayneh et al. 2020)	Oral hygiene	PI	1.88	1.27	N/A	N/A	
							Significant improvements in PI and GI
		GI	1.12	0.85	N/A	N/A	
		DMFT/dmft	3.78	NR	N/A	N/A	
(Sallam et al. 2013)	Behavior	Easily accepted brushing	B: 8.3% C: 0%	B: 58.3% C: 41.7%	A: 0%	A: 16.66%	Video Modeling (C) was the most effective method for improving children's behavior toward oral care. Pictorial Activity Schedules (B) were also effective but required more time for acquisition compared to video modeling. Demonstration modeling (A) alone was not sufficient to bring about significant change in behavior.
(Krishnan et al. 2021)	Oral hygiene	PI	2.02 ± 0.06	0.45 ± 0.13	2.01 ± 0.06	0.46 ± 0.11	Statistically significant improvements in PI and GI
		GI	1.05 ± 0.13	0.28 ± 0.10	1.03 ± 0.17	0.24 ± 0.11	
	Perception and practice	Perception and practice					Significant improvement in parental perception and oral hygiene practice
(Pai Khot et al. 2023)	Ability	Practice Score	2.53 ± 1.25	5.50 ± 1.12	2.47 ± 1.38	3.57 ± 1.10	Improvement in oral hygiene practices
	Perception	Perception					Improvement in Caregiver's oral hygiene perception
	Oral hygiene	OHI‐S	2.18 ± 0.28	1.22 ± 0.14	2.05 ± 0.30	1.94 ± 0.15	A statistically significant improvements in PI and GI
		GI	1.22	0.35 ± 0.12	1.39	0.83 ± 0.37	
(Du et al. 2021)	Oral hygiene	PI	1.00 ± 0.32	0.63 ± 0.25	N/A	N/A	Significantly improved oral hygiene's indices; specially in children with poorer baseline oral health
		Percentage of Sites with Plaque	84% ± 20	61% ± 23			
		GI	0.91 ± 0.26	0.60 ± 0.26			
		Percentage of Sites with Gingivitis	83% ± 21	58% ± 24			
(Mahajan et al. 2023)	Oral hygiene	PI	Males:2.08 ± 0.01, Females: 1.98 ± 0.05	Males: 0.23 ± 0.05, Females: 0.19 ± 0.12	N/A	N/A	Statistically significant improvements in PI
(Pawar et al. 2022)	Oral hygiene	OHI‐S	1.46 ± 0.47	0.43 ± 0.47	1.54 ± 0.52	1.15 ± 0.49	Statistically significant improvements in OHI‐S
(Carli et al. 2022)	Oral hygiene	Brushing Frequency	2.29 ± 0.85	2.88 ± 0.35	N/A	N/A	Increased brushing frequency and reduced frequency of snacks
		Frequency of Snacks	1.05 ± 0.67	0.87 ± 0.46			
		PI	3.20 ± 0.76	1.41 ± 0.55			Statistically significant improvements in PI and GI
		GI	2.03 ± 0.80	1.34 ± 0.80			
		DMFT/dmft	0.11 ± 0.14	0.15 ± 0.14			No significant change in DMFT/dmft
	Behavior	Frankl Scale	2.04 ± 0.75	3.20 ± 0.66			Improvement in Frankl Scale
(Doichinova et al. 2019)	Oral hygiene	OHI‐S	2.29	1.79	N/A	N/A	Statistically significant improvements in OHI‐S
(Fenning et al. 2022)	Oral hygiene	Frequency of Twice‐daily brushing	40%	78%	40%	62%	better long‐term adherence to twice‐daily brushing routines
		PI	0.84 ± 0.35	0.75 ± 0.5	0.84 ± 0.35	0.80 ± 0.5	Significant improvements in oral health indices
		dmft/DMFT	0.88	0.93	0.88	1.09	
		No. Decayed tooth	0.29	0.34	0.29	0.51	
	Behavioral	Behavior problems (CBCL)	2.89	1.37	2.89	2.14	Easier and less stressful home oral hygiene routines and reduced child resistance to toothbrushing
(Shalabi et al. 2022)	Oral hygiene	OHI‐S	3.45 ± 0.28	1.97 ± 0.55	3.56 ± 0.30	2.54 ± 0.39	A statistically significant improvement in OHI‐S
		Simplified DI	2.17 ± 0.14	1.28 ± 0.26	2.24 ± 0.18	1.62 ± 0.23	
		Simplified CI	1.28 ± 0.14	0.69 ± 0.29	1.32 ± 0.12	0.92 ± 0.16	
(Renuka et al. 2022)	Oral hygiene	OHI‐S	2.566 ± 0.504	1.800 ± 0.667	N/A	N/A	A significant improvement in OHI‐S
(Piraneh et al. 2023)	Oral hygiene	OHI‐S	1.85 ± 2.26	1.24 ± 0.34	1.98 ± 0.54	1.48 ± 0.50	A significant improvement in OHI‐S
	Parents knowledge and attitude	Parents' oral health knowledge	85.71 ± 15.14	91.78 ± 12.50	86.84 ± 14.06	93.53 ± 12.15	There was no significant difference in oral health behaviors between the groups, both groups showed an improvement in oral health knowledge and attitude post‐intervention.
		Parents' oral health attitude	83.34 ± 13.50	87.77 ± 11.73	82.03 ± 12.51	87.43 ± 8.43	
		Daily brushing frequency (Once or more)	68.4%	76.6.%	58.6%	69.6%	
		Routines of tooth‐brushing (Unaided)	12.7%	9.1%	17.2%	12.5%	
		Use of fluoridated tooth paste (Most of the time)	75.9%	83.1%	74.1%	82.1%	
(Zhou et al. 2020)	Oral hygiene	dmft	1.4 ± 2.7	0.8 ± 1.3	1.2 ± 2.5	0.7 ± 1.4	No significant difference in dmft
		Modified GI	1.2 ± 0.6	0.2 ± 0.2	1.3 ± 0.6	0.4 ± 0.4	A statistically significant improvement in oral hygiene indices
		DI‐S	1.8 ± 0.8	0.4 ± 0.4	1.6 ± 0.6	0.7 ± 0.5	
		Toothbrushing Steps	6.5 ± 3.0	10.7 ± 2.7	6.9 ± 4.0	9.2 ± 3.6	More toothbrushing steps and spent more time brushing teeth.
		Toothbrushing Duration	91.6 ± 58.3	170.4 ± 60.4	92.7 ± 64.3	148.9 ± 69.9	
	Behavior	Children Who Visited a Dentist	19.2%	28.8%	18.0%	18.7%	Higher rates of annual dental visits
(Pilebro and Bäckman 2005)	Oral hygiene	Abundant Plaque	8 children	3 children	N/A	N/A	A significant improvement in PI
		Visible Plaque	6 children	7 children			
		No Visible Plaque	0 children	4 children			
	Parental perception of difficulty	Very Difficult	9 parents	2 parents			Easier toothbrushing
		Difficult	5 parents	4 parents			
		Easy	0 parents	8 parents			
(Lefer et al. 2018)	Autonomy	Overall Brushing Autonomy (Mean Score)	3.7 ± 1.0	4.1 ± 0.7	N/A	N/A	A significant improvement in autonomy in all brushing steps (preparation, brushing, and finishing).
(Smutkeeree et al. 2020)	Behavior	Toothbrushing Ability	0.6 ± 0.5	2.4 ± 0.8	N/A	N/A	Significant improvements in toothbrushing ability and cooperation
		Toothbrushing Cooperation	2.4 ± 0.8	3.5 ± 0.7			
	Oral hygiene	Plaque Index Score	1.8 ± 0.7	0.6 ± 0.3			A significant improvement in PI.
(Lopez Cazaux et al. 2019)	Autonomy	Toothbrushing Autonomy Score (SAut)	3.2 ± 0.9	4.2 ± 0.7	N/A	N/A	A significant improvement in brushing autonomy
		Final Steps Score (SEnd)	3.9 ± 0.9	4.3 ± 0.8			
(Chawla and Goswami 2021)	Oral hygiene	PI	2.04 ± 0.65	2.00 ± 0.33	N/A	N/A	A significant improvement in oral hygiene indices
		Mean Toothbrushing Time	1.01 ± 0.57	1.6 ± 0.09			
		Children Brushing Twice Daily (%)	20%	60%			
		Children Requiring Assistance (%)	100%	50%			
(Ramassamy et al. 2019)	Oral hygiene	PI	1.75 ± 0.25	0.96 ± 0.34	1.78 ± 0.14	1.35 ± 0.35	A statistically significant improvement in oral hygiene indices and reduced resistance to brushing in the yoga group
		GI	1.72 ± 0.22	1.09 ± 0.27	1.76 ± 0.14	1.49 ± 0.18	

Abbreviations: CBCL, Child Behavior Checklist; CI, calculus index; DI, debris index; DMFT/dmft, decayed, missing due to caries, and filled permanent/deciduous teeth; GI, gingival index; N/A, not applicable; OHI‐S, Simplified Oral Hygiene Index Score; PCR, polymerase chain reaction; PI, plaque index.

For children's interventions, in the Visual channel, seven studies (Gandhi et al. 2024; Popple et al. 2016; Krishnan et al. 2021; Mahajan et al. 2023; Carli et al. 2022; Lefer et al. 2018; Lopez Cazaux et al. 2019) investigated Email and application interventions, seven studies (Chawla and Goswami 2021; Gandhi et al. 2024; Popple et al. 2016; Mahajan et al. 2023; Sallam et al. 2013; Shalabi et al. 2022; Ramassamy et al. 2019) investigated Video, and 12 studies (Pai Khot et al. 2023; Popple et al. 2016; Krishnan et al. 2021; Sallam et al. 2013; Shalabi et al. 2022; Ramassamy et al. 2019; Al‐Batayneh et al. 2020; Doichinova et al. 2019; Renuka et al. 2022; Zhou et al. 2020; Pilebro and Bäckman 2005; Smutkeeree et al. 2020) investigated Pictures/Images; three studies (Pai Khot et al. 2023; Gandhi et al. 2024; Sallam et al. 2013) investigated the Verbal channel, of which one study was not significant (Sallam et al. 2013). One study used a social story and a Standard Leaflet (Zhou et al. 2020). Three studies (Ramassamy et al. 2019; Vajawat et al. 2015; Pawar et al. 2022) investigated oral health procedures, including powered or manual tooth brushing (Vajawat et al. 2015; Pawar et al. 2022) and Yoga therapy as an adjunctive intervention (Ramassamy et al. 2019).

For parents/caregivers/teachers interventions, in the Visual channel, one study (Mahajan et al. 2023) investigated Email and application interventions, three studies (Du et al. 2022b; Mahajan et al. 2023; Piraneh et al. 2023) investigated Video, and eight studies investigated Pictures/Images (Du et al. 2022b; Al‐Batayneh et al. 2020; Doichinova et al. 2019; Zhou et al. 2020; Pilebro and Bäckman 2005; Piraneh et al. 2023; Aljubour et al. 2022; Du et al. 2021); three studies investigated the Verbal channel (Fenning et al. 2022; Du et al. 2022b; Ibrahim et al. 2020); two studies used a booklet/Leaflet (Mah et al. 2023) and one study investigated oral health procedures, which include DentalToolKit (Fenning et al. 2022).

These studies not only reported oral health indices such as PI, DI, CI, and OHI‐S, GI, and DMFT; but also, behavioral changes such as autonomy, satisfaction, acceptance, Frankl score, and Cooperation, performance and ability and also changes in parent's knowledge, attitude and practice.

### 3.4 Risk of Bias Assessment

**Figure 2:**
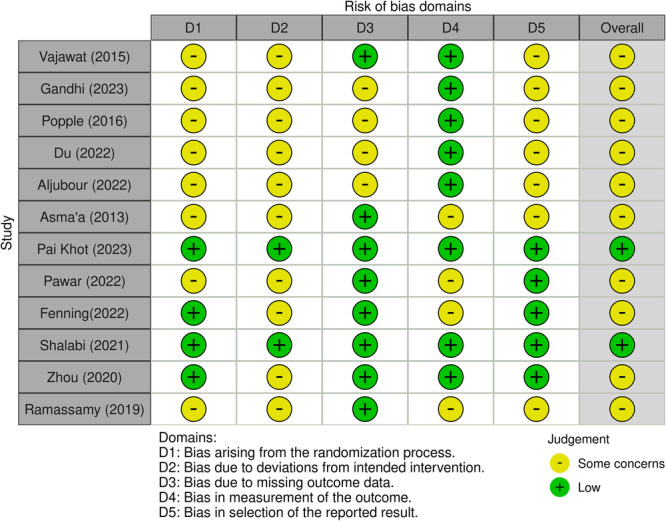
Risk of bias assessment of RCTs using the ROB‐2 tool.

**Figure 3:**
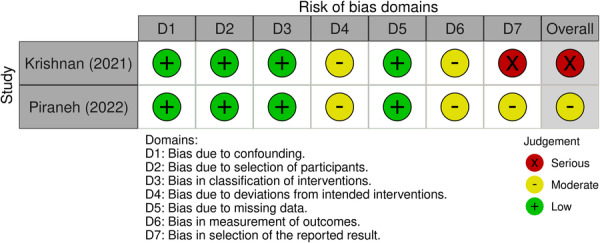
Risk of bias assessment of nonrandomized studies of intervention (NRSI) using the ROBINS‐I tool.

**Figure 4:**
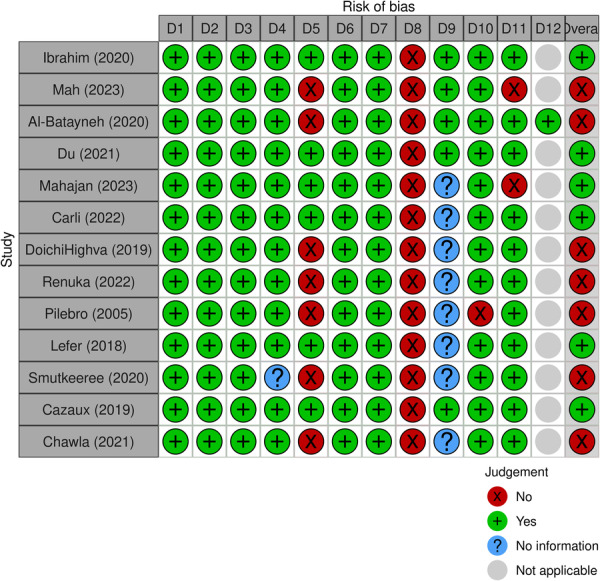
Risk of bias assessment of quasi‐experimental studies using NIH tool.

Regarding the 12 RCTs (Figure 2), two were judged at a low risk of bias (Pai Khot et al. 2023; Shalabi et al. 2022) and others at a moderate risk of bias (Fenning et al. 2022; Du et al. 2022b; Gandhi et al. 2024; Popple et al. 2016; Sallam et al. 2013; Ramassamy et al. 2019; Zhou et al. 2020; Vajawat et al. 2015; Pawar et al. 2022; Aljubour et al. 2022); among the two non‐randomized studies (Figure 3), one was judged at a moderate risk of bias (Piraneh et al. 2023), and the other one was at a serious risk of bias (Krishnan et al. 2021). Also, the risk of bias assessment among 13 quasi‐experimental studies showed that seven studies were judged poor (Chawla and Goswami 2021; Al‐Batayneh et al. 2020; Doichinova et al. 2019; Renuka et al. 2022; Pilebro and Bäckman 2005; Smutkeeree et al. 2020; Mah et al. 2023), and the others were judged good (Mahajan et al. 2023; Carli et al. 2022; Lefer et al. 2018; Lopez Cazaux et al. 2019; Du et al. 2021; Ibrahim et al. 2020) (Figure 4).

The GRADE framework rated the strength of the evidence for all outcomes as Low.

#### 3.4.1 Stratified Meta‐Analysis

**Figure 5:**
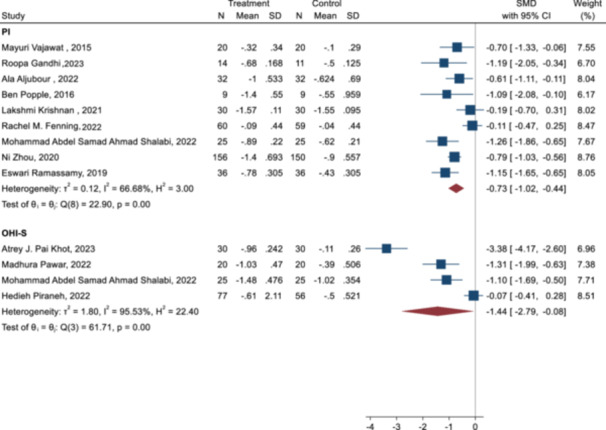
Forest plot of the effect of interventions on OHI‐S and PI indices.

The random‐effect meta‐analysis according to type of index showed that the interventions was significant for PI (SMD = −0.73 [−1.02 to 0.44]) and OHI‐S index (SMD = −1.44 [−2.79 to −0.08]) (Figure 5). The result of the random model showed that interventions were effective on combination of both indices (OHIS and PI) (SMD = −0.95 [−1.39 to −0.52]).

The results of the meta‐analysis and subgroup analysis are reported in Table 4. There were 12 studies (13 points of data) for combination of OHI‐S and PI indices.

**Table 4:** Stratified and sensitivity analysis of meta‐analysis findings.

Outcome	No. of studies	Sample size	Pooled SMD (95% CI)	Heterogeneity assessment		
				*I*‐squared (%)	Model	*p* value
Plaque indices (PI and OHI‐S)	12^a^	I: 511 C: 480	−0.95 (−1.39 to −0.52)	90.24	Random	< 0.001
PI	9	I: 382 C: 372	−0.73(−1.02 to −0.44)	66.68	Random	< 0.001
OHIS	4	I: 154 C: 133	−1.44 (−2.79 to −0.08)	95.53	Random	< 0.001
Randomized studies	11	I: 402 C: 392	−1.12 (−1.65 to −0.65)	88.56	Random	< 0.001
Nonrandomized studies	2	I: 109 C: 88	−0.11 (−0.39 to 0.18)	0	Fixed	0.68
Video‐based interventions	5^a^	I: 157 C: 131	−0.69 (−1.2 to −0.14)	80.38	Random	< 0.001
GI (all studies)	6	I: 286 C: 277	−0.74(−1.34 to −0.14)	88.72	Random	0.01
GI (excluding nonrandomized study)	5	I: 256 C: 247	−0.92 (−1.52 to −0.32)	85.79	Random	< 0.001

Abbreviations: GI, gingival index; OHI‐S, Simplified Oral Hygiene Index Score; PI, Plaque index.

^a^ One study reported both OHI‐S and PI (12 studies and 13 points of data).

Also, stratified meta‐analysis based on the mode of intervention showed that video intervention significantly improves PI compared to other modes of intervention (SMD = −0.69 [−1.2 to −0.14] with obvious heterogeneity (*Q* = *26.63, P* < *0.001, I‐square* = *80.38*) (Table 4).

**Figure 6:**
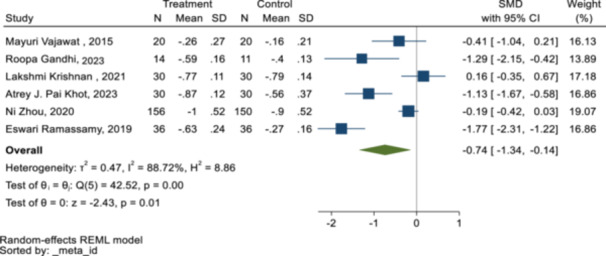
Forest plot of the effect of interventions on GI.

Moreover, there was a significant improvement of GI index (SMD = −0.74 [−1.34 to −0.14] with significant heterogeneity (*Q* = *88.72, p = 0.0, I‐square* = *45.52*) (Figure 6).

#### 3.4.2 Publication Bias

The results of Egger's test did not support the existence of publication bias for the GI test (*p* = 0.29; coefficient = −3.31).

Egger's test showed evidence of significant publication bias for OHI‐S (*p* = 0.017; coefficient = −4.37). The trim and fill analysis showed that five missing studies were identified. After imputing these studies, the adjusted SMD of OHI‐S was estimated at −1.386 [−1.843 to −0.929].

#### 3.4.3 Sensitivity Analysis

Sensitivity analysis was performed to assess the effect of excluding non‐randomized studies on the pooled effect size. After exclusion of non‐randomized studies (two studies), the pooled effect size for PI did not change significantly and remained statistically significant (SMD = −1.12 [−1.58 to −0.65]) (Table 4 *)*.

For the GI index, after the exclusion of one non‐randomized study, the pooled SMD changed from (−0.74 [− 1.34 to −0.14]) to (−0.92 [−1.52 to −0.32]) (Table 4).

## 4 Discussion

To the best of our knowledge, this is the first systematic review and meta‐analysis assessing all interventions that promote oral health in children with ASD. The main goal of the present study was to document and compare the effectiveness of oral health interventions. The interventions have been proposed as an effective approach to promote oral health indices in children with ASD. Also, these methods help them improve oral health behavior. Studies also showed that parents’ involvement can be effective.

A meta‐analysis of these studies showed that multiple modes of intervention can improve oral health outcomes including OHI‐S, PI, and GI indices. Mixed‐method analysis showed that the DMFT/dmft as a dental index was improved by educational interventions include visual, verbal, and booklet/leaflet. Studies also show that e‐mails, applications, videos, and pictures are effective in visual interventions. Procedures and activities such as yoga and powered tooth brushing can also be helpful.

Our findings showed that video‐based interventions are more effective than other interventions (pictures and verbal) in improving oral health outcomes such as OHI‐S and PI. This finding was concordant with previous prior studies (Gandhi et al. 2024; Popple et al. 2016; Krishnan et al. 2021; Shalabi et al. 2022; Balian et al. 2021) that highlight the efficacy of video‐based approaches in ASD populations.

Almost all studies investigating behavior during dental care showed increased children's cooperation. Overall, visual pedagogy improves oral hygiene/tooth brushing skills and cooperation levels. Children's behavioral feedback, such as autonomy, acceptance, satisfaction, performance, cooperation, and tooth brushing ability, improves through some interventions.

Studies also showed that visual education can improve Frankl's score; however, verbal education is effective in oral health‐related QALY (OHRQoL). Also, one study showed that storytelling is more effective in visual pedagogy than non‐storytelling pictures.

Parents' involvement is associated with oral health and children's behavior. Interventions can improve parents’ knowledge, attitudes, and practices (KAP), aligning with the KAP model (Tahani et al. 2022) for oral health interventions. These interventions include using guideline booklets, verbal or demonstration for them, or participating them in oral health activities, or using media for children.

This study's strengths lie in its systematic and accurate approach, adhering to PRISMA guidelines for data synthesis. By including all available studies on oral health interventions for children with ASD, we ensured a comprehensive analysis of the current evidence base. However, as a secondary study, our analysis is inherently limited by the quality and availability of primary data. Variability in study designs, sample sizes, and intervention protocols, different intervention groups and different comparators, and a lack of sufficient studies cause challenges in pooling results; however, subgroup analyses were performed to mitigate this issue. Quality assessment tools were employed to evaluate study quality, while publication bias was evaluated, enhancing the strength of our findings. Quality assessment (low to moderate) and GRADE framework (low for all outcomes), and also heterogeneous study protocols, show that further studies are needed to improve the evidence. The primary source of bias was blinding, which should be considered in future studies.

The implications of our findings are far‐reaching and hold significant relevance for various stakeholders. For clinicians, the evidence supports the integration of visual pedagogy and parental involvement into routine dental care for children with ASD, thereby enhancing oral hygiene and cooperation. Parents can benefit from accessible educational tools like booklets and media‐based resources to improve their knowledge, attitude, and behavior and support their children's oral health. Policymakers should consider incorporating these evidence‐based interventions into public health programs to address oral health disparities in ASD populations. For researchers, our review underscores the need for standardized outcome measures and high‐quality, large‐scale randomized controlled trials to elucidate the comparative effectiveness of intervention modalities further. Additionally, exploring the long‐term sustainability of these interventions and their impact on quality of life will be critical for advancing care in this population.

## 5 Conclusion

This systematic review and meta‐analysis, confirms that visual pedagogy significantly improved oral health in children with ASD. Video‐based interventions outperform traditional methods like verbal or picture‐based approaches. Parental engagement amplifies these outcomes by improving knowledge, attitudes, and practices.

## Author Contributions

Azadeh Babaei and Mostafa Qorbani proposed the concept of this study. Shirin Djalalinia, Mostafa Qorbani, and Azadeh Babaei designed the study. Iman Rajaei and Azadeh Babaei performed the data extraction. Mostafa Qorbani, Azadeh Babaei, and Iman Rajaei conducted the analysis and interpretation. Shirin Djalalinia carried out the literature search. Iman Rajaei and Azadeh Babaei conducted the screening. Iman Rajaei and Azadeh Babaei wrote the study. All authors read and approved the final manuscript.

## Funding

The authors received no specific funding for this work.

## Conflicts of Interest

The authors declare no conflicts of interest.

## Data Availability

Data available upon reasonable request from the corresponding author.
